# Impaired Lower Limb Proprioception in Spinocerebellar Ataxia Type 3 and Its Affected Factors

**DOI:** 10.3389/fneur.2022.833908

**Published:** 2022-02-02

**Authors:** Xia-Hua Liu, Zhi-Yong Wang, Ying Li, Hao-Ling Xu, Arif Sikandar, Jun Ni, Shi-Rui Gan

**Affiliations:** ^1^Department of Rehabilitation Medicine, The First Affiliated Hospital, Fujian Medical University, Fuzhou, China; ^2^The Third Clinical Medical College, Fujian Medical University, Fuzhou, China; ^3^Department of Neurology, Institute of Neurology, The First Affiliated Hospital, Fujian Medical University, Fuzhou, China

**Keywords:** spinocerebellar ataxia type 3, lower limb proprioception, Pro-kin system, postural control, disease progression

## Abstract

**Background:**

Spinocerebellar ataxia type 3 (SCA3) is one of the most common hereditary neurodegenerative diseases. Postural control dysfunction is the main symptom of SCA3, and the proprioceptive system is a critical sensory component of postural control. Accordingly, proprioception quantification assessment is necessary in monitoring the progression of SCA3.

**Objective:**

We aimed to quantitatively assess lower limb proprioception and investigate the relationship between proprioception and clinical characteristics in patients with SCA3.

**Methods:**

A total of 80 patients with SCA3 and 62 health controls were recruited, and their lower limb proprioception was measured using the Pro-kin system. Clinical characteristics of the SCA3 patients were collected. Multivariable linear regression was used to investigate potential affected factors for lower limb proprioception.

**Results:**

We found that the patients with SCA3 experience poorer lower limb proprioception characterized by significant impairment in the average trace error (ATE) and time to carry out the test time execution (TTE) compared to controls (*P* < 0.05). Moreover, there were significant differences in TTE between the right and left lower limbs (*P* < 0.05) of the patients. Regression analyses revealed that increasing age at onset (AAO) predicts poorer lower limb proprioception for both ATE (β = 2.006, *P* = 0.027) and TTE (β = 1.712, *P* = 0.043) and increasing disease duration predicts poorer lower limb proprioception for ATE (β = 0.874, *P* = 0.044). AAO (β = 0.328, *P* = 0.019) along with the expanded alleles (β = 0.565, *P* = 0.000) could affect the severity of ataxia. By contrast, ATE (β = 0.036, *P* = 0.800) and TTE (β = −0.025, *P* = 0.862) showed no significant predictors.

**Conclusions:**

Lower limb proprioception in patients with SCA3 is significantly impaired when compared to healthy controls. Increasing AAO and disease duration are related to impaired lower limb proprioception.

## Introduction

Spinocerebellar ataxia type 3 (SCA3), also known as Machado-Joseph disease (MJD), is the most common inherited spinocerebellar ataxias and one of autosomal dominant neurodegenerative disorders with high clinical heterogeneity (1). SCA3 is caused by the expansion of cytosine-adenine-guanine (CAG) triplet repetitions in the coding region of the ATXN3 gene (14q32.1), which results in an expanded polyglutamine repeat in the encoded ataxin-3 protein, causing severe atrophy of the cerebellar Purkinje cell layer, brain stem, cortex and spinal cord (2, 3). The clinical characteristics of patients with SCA3 have various manifestations, including postural control dysfunction, gait ataxia, oculomotor abnormalities, dysarthria and peripheral neuropathy (4).

Postural control dysfunction, one of the main factors of SCA3 directly affecting gait, is associated with an increased incidence of falls in this population (5–7). Postural control is a complex process requiring the central integration of numerous sensory-motor processes (8). The influence of motor control and vision aspects on postural control has been reported in SCAs (9, 10). The proprioceptive system is a critical sensory component of postural control (11). It has been suggested that cerebellar damage may cause varied impairments to proprioceptive sense (12). Therefore, pathological processing of proprioceptive information may be a key pathological mechanism of postural control dysfunction in SCA3. Several studies about other neurological disorders, such as stroke, multiple sclerosis, Parkinson's disease, and Huntington's disease, have suggested that disturbances in proprioception are important for the postural control of patients (13–15). However, little attention has been directed toward proprioceptive deficits in the progression of SCA3, which is the most common subtype of SCAs (16).

Pro-kin system [Prokin 254 (Pro-Kin Software Stability), TecnoBody S. r. l., Dalmine, 24044 Bergamo, Italy] is an advanced evaluation technology with a computerized proprioceptive kinematic footboard. It can analyze and integrate the on-screen tracks of patients to generate targeted assessment and rehabilitation paths of proprioceptive deficits (15). The validity of Pro-kin to assess postural instability in patients with SCA3 has been demonstrated (17), and the system has been used to test lower limb proprioception function in patients with hemiparetic stroke, multiple sclerosis, total knee prosthesis and knee osteoarthritis (15, 18, 19). Our previous research used the Pro-kin system to evaluate the postural control stability of SCA3 patients and showed that postural instability may be correlated with disease severity (17). Using the Pro-kin system to assess the postural control dysfunction of advancing neurodegenerative diseases, including SCA3, can become a potential longitudinal biomarker. However, as one component of postural control, proprioception in SCA3 is barely studied, and the relationship between proprioception and the clinical characteristics of patients with SCA3 remains unclear.

In this study, we intend to use the Pro-kin system to quantitatively identify lower limb proprioception in SCA3 and explore the correlation between lower limb proprioception and the clinical characteristics of patients with SCA3 in order to formulate a rehabilitation treatment plan for SCA3 for reference.

## Methods

### Participants

Employing a cross-sectional design, this study included both individuals with a diagnosis of molecular-confirmed SCA3 (20) and healthy controls (HCs) as participants. Between October 2018 and December 2019, we recruited 80 patients with SCA3 and 62 HC participants at The First Affiliated Hospital of Fujian Medical University in Fuzhou. The protocol was approved by the Ethics Committee of The First Affiliated Hospital, Fujian Medical University [Approval No: MRCTA, ECFAH of FMU(2018)201]. The design and procedures of the study were performed in accordance with the Declaration of Helsinki. Written informed consent was obtained from the participants prior to their participation.

The inclusion criteria for participants with SCA3 were (1) a definite genetic diagnosis of SCA3; (2) 20–80 years of age; (3) Mini-Mental State Examination score > 27; (4) ability to stand independently in the upright position for 30 s; (5) no evidence of other neurological, musculoskeletal or cardiovascular disorders; and (6) a willingness to participate. The exclusion criteria were (1) unstable vital signs and uncontrolled hypertension; (2) history of vestibular symptoms or vestibular disease; (3) presence of cognitive impairment, visual or hearing pathologies; (4) inability to stand independently for 30 s with eyes closed; (5) lack of sensitivity in the lower limbs; (6) musculoskeletal, cardiovascular or respiratory system impairments or other accompanying ailments; (7) engagement in another rehabilitative study protocol; (8) peripheral neuropathy; or (9) on medication affecting the musculoskeletal system or proprioception and postural stability (e.g., anti–depressants, dopaminergic agents, hypnotics).

The control group, which included mostly spouses and caregivers of patients with SCA3/MJD, was matched for age, gender and environmental characteristics. Relatives at risk were excluded from the HC group. The control group comprised 62 individuals without neurological, musculoskeletal or cardiorespiratory impairments.

### Genotype and Phenotype Analysis

Genomic DNA was extracted from peripheral blood samples provided by each patient using QIAamp DNA Blood Mini kit (Qiagen, Hilden, Germany). The numbers of CAG repeats of the patients were determined by polymerase chain reaction amplification combined with Sanger sequencing, as previously reported (20).

Ataxia specialists interviewed each patient to obtain all information needed for the present study. Age at onset (AAO) was defined as the age when ataxia symptoms related to SCA3 first appeared, which was estimated according to the reports of patients, close relatives, or care providers. Disease duration was the time span between AAO and the age at first visit. The severity of ataxia was assessed with the Scale for the Assessment and Rating of Ataxia (SARA), which comprised of eight cerebellar function tests with a total score ranging from 0 (absence of ataxia) to 40 (most severe ataxia) (21).

### Pro-kin System Assessment

**Figure 1:**
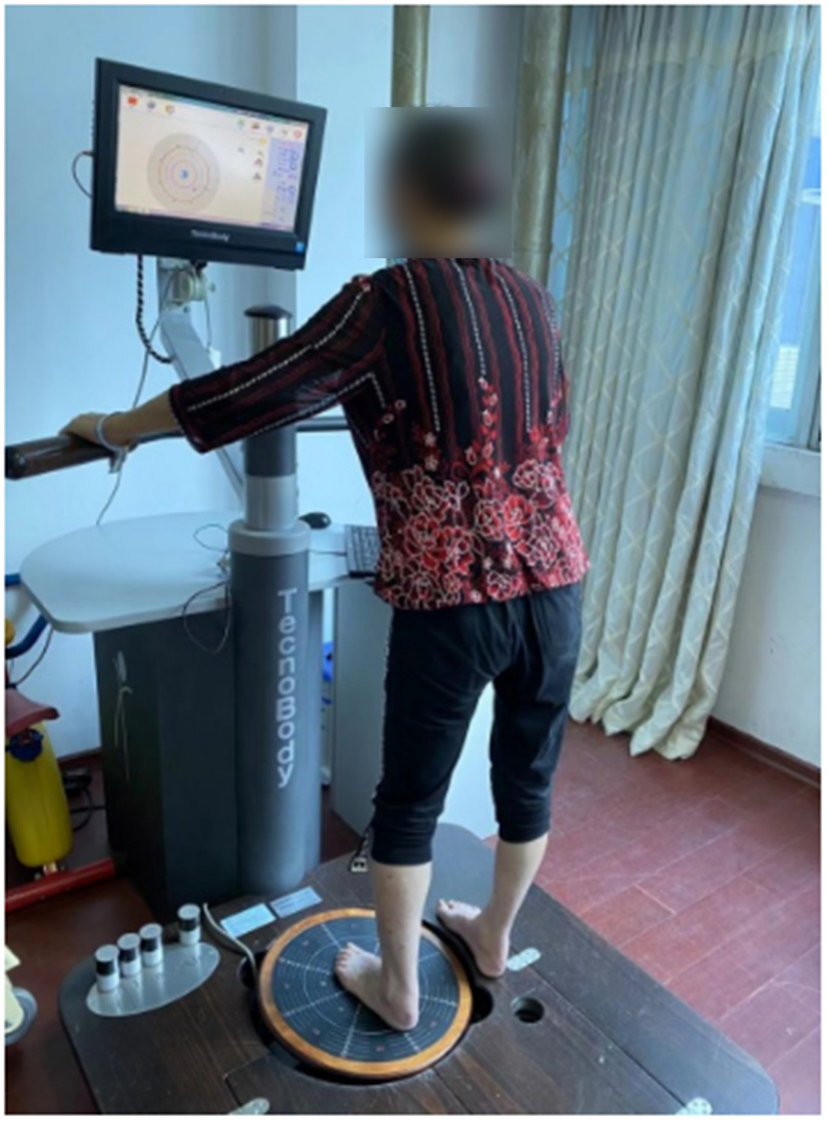
Participant used the Pro-kin system to test the lower limb proprioception.

We conducted an observational study between SCA3 patients and HC participants at the same time of the day. Pro-kin system was used to measure the proprioception on a multiaxial balance evaluator for lower limbs. A rehabilitation therapist blinded to the groups conducted the assessments. All participants received the assessments in a quiet and naturally and brightly lit room. Participants are evaluated as shown in Figure 1.

**Figure 2:**
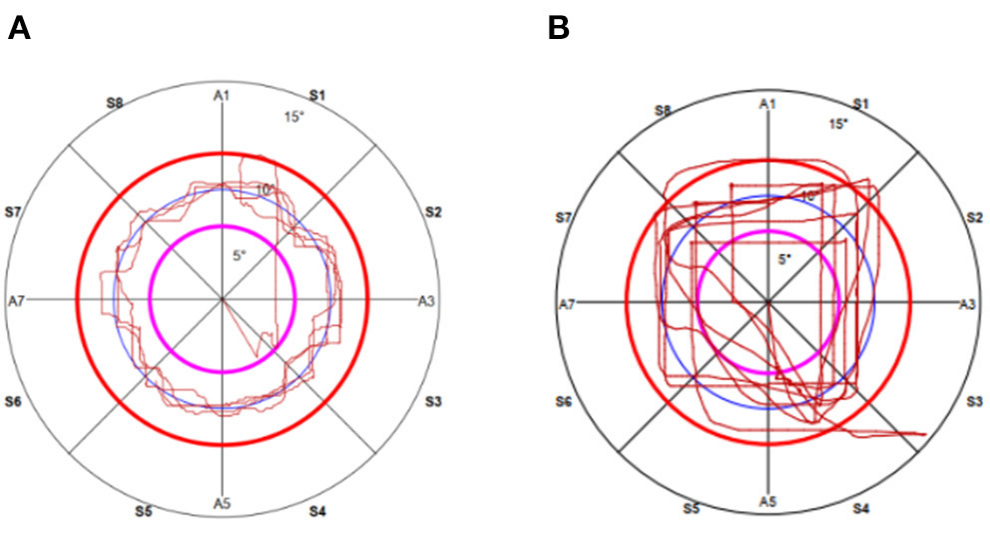
The actual recording of the path of circular during proprioceptive multiaxial assessment in the right lower limb in **(A)** a health control subject and **(B)** a SCA3 patient.

To measure lower limb proprioception, the footboard of the system was set to allow angular movements in the sagittal and frontal planes. Participants placed their right limb on the footboard and then their left limb on a fixed support platform equal in height to the footboard (15). They were then asked to draw circular route lines on the screen by moving their lower limb (on the footboard). Their motor task was to try to keep the circular route lines drawn by the movements of their lower limb (on the footboard) superimposed as much as possible on those already drawn by the system (Figure 2). The test stopped automatically at the end of five turns. The right lower limb was measured first and then the left lower limb. Average trace error (ATE) and time to carry out the test time execution (TTE) [s] were evaluated (15, 18, 19). Large ATE values mean large errors in path control, which indicate poor lower limb proprioception. TTE was the time from the start to the end of the trial.

Each test was repeated three times and the mean scores were recorded. The assessment lasted approximately 20 minutes. The participants were oriented to the test in the evaluation mode before performing the actual test. To reduce the risk of falls and minimize interference from external support, a trainer stayed close alongside or behind the participants.

### Statistical Analyses

For analyses of the basic demographics between SCA3 patients and HC subjects, Chi-square tests were used to compare the gender distribution. Two independent samples T-tests and Mann-Whitney U tests test were used, respectively, for normal and non-normal distributed variables, the result of which were confirmed by Kolmogorov-Smirnov testing. Following the results of these tests, variables with normal distribution and non-normal distribution were expressed as mean ± SD and median (range), respectively.

We used multivariable linear regression to examine the relationship between proprioception and phenotype variability in patients with SCA3. First, we assessed the predictors of proprioception measures: ATE and TTE. These measures were predicted by the following independent variables: gender (binary), AAO, disease duration, SARA score and length of normal and expanded CAG repeats. Next, we analyzed the predictors of the severity of ataxia as measured with the SARA score by using the following independent variables: ATE, TTE, gender (binary), AAO and length in normal and expanded CAG repeats.

All the statistical analyses were performed using SPSS version 20.0 (SPSS Inc., Chicago, IL, USA). The results were considered statistically significant at *P* < 0.05.

## Results

Table 1 shows the demographic characteristics of 80 patients with SCA3 (male: 43, female: 37) and 62 healthy control participants (male: 31, female: 31). There were no statistically significant sociodemographic (i.e., age and gender) differences between patients with SCA3 and the HC group. In the patients, the mean AAO was 32.34 ± 8.63 years, average disease duration was 7.10 ± 3.56 years, median length of expanded alleles was 75 (69–81), median length of CAG repeats in normal alleles was 19 (13–44) and average SARA score was 9.23 ± 2.86.

**Table 1:** Demographic characteristics of study subjects.

	**SCA3 group**	**HC group**	***P-*value**
Sample size, *N*	80	62	NA
Age, years	39.14 ± 9.58	42 (18–65)	0.594^a^
Gender (male/female)	43/37	31/31	0.657^b^
Dominant side (right/left)	Right	Right	NA
Disease duration, years	7.10 ± 3.56	NA	NA
Age at onset, years	32.34 ± 8.63	NA	NA
Normal alleles, *N*	19 (13–44)	NA	NA
Expanded alleles, *N*	75 (69–81)	NA	NA
SARA score	9.23 ± 2.86	NA	NA

*SARA, The Scale for the Assessment and Rating of Ataxia; N, number; NA, not applicable.*

*Variables with normal distribution were represented as mean ± standard deviation; variables in non-normal distribution were expressed as median (range).*

*^a^Mann-Whitney U-test.*

*^b^Chi-square test*.

There are two groups of lower limb proprioception outcome variables presented in Table 2. We first compared all outcome variables separately for the right and left limbs between the patient and control groups. We found that ATE on the left limb and TTE on both lower limbs were significantly worse for patients compared to controls (*P* < 0.05). Second, we compared all outcome variables separately for the patient and control groups between the right and left limbs. We found that though both groups have bigger outcomes in ATE and TTE in the left limb compared to the right limb, while there was only a significant difference in TTE between their right and left limbs in SCA3 patients (*P* < 0.05).

**Table 2:** Lower limb proprioception measures in SCA3 and health controls.

**Indices**	**Lower limb**	**SCA3 group**	**HC group**	***P-*value**
ATE	R	33 (8.66–124.33)	26 (3.67–101.33)	0.066^a^
	L	53.66 ± 29.43	29.67 (3.00–124.00)	**0.035** ^**a**^
	*P-*value	0.142^a^	0.192^a^	
TTE	R	100 (73.67–202)	76(0–152.67)	**0** ^**a**^
	L	100.66(0–205.33)	79.67(61.67–154.00)	**0** ^**a**^
	*P-*value	**0.042** ^**a**^	0.327^a^	

*ATE, Average Tace Error; TTE, Test Time Execution; R, Right; L, Left.*

*Variables with normal distribution were represented as mean ± standard deviation; variables in non-normal distribution were expressed as median (range). Bold value showed significance.*

*^a^Mann-Whitney U-test*.

Multivariable linear regression was used to examine the relationship between lower limb proprioception measures and clinical characteristics in patients. First, we investigated the predictors of lower limb proprioception (Table 3) using regression models predicting ATE and TTE. The results indicated that increasing AAO predicts poorer lower limb proprioception for both ATE (β = 2.006, *P* = 0.027) and TTE (β = 1.712, *P* = 0.043) and increasing disease duration predicts poorer lower limb proprioception for ATE (β = 0.874, *P* = 0.044). On the contrary, higher lengths of CAG repeats in expanded alleles do not predict poorer lower limb proprioception for both ATE (β = −0.200, *P* = 0.301) and TTE (β = −0.159, *P* = 0.376). Next, we also performed multivariable linear regression to examine whether lower limb proprioception could affect the severity of ataxia (Table 4). We found that AAO (β = 0.328, *P* = 0.019) along with the expanded alleles (β = 0.565, *P* = 0.000) could affect the severity of ataxia. By contrast, ATE (β = 0.036, *P* = 0.800) and TTE (β = −0.025, *P* = 0.862) showed no significant predictors.

**Table 3:** The affected factors on lower limb proprioception.

	**Coefficient estimate**	**Standard error**	***P-*value**
**ATE**			
Gender^a^	0.081	6.368	0.547
AAO	2.006	2.409	**0.027**
Disease duration	0.874	2.864	**0.044**
SARA	0.122	2.498	0.685
Normal alleles	−0.039	0.445	0.771
Expanded alleles	−0.200	1.616	0.301
**TTE**			
Gender^a^	0.074	7.007	0.557
AAO	1.712	2.651	**0.043**
disease duration	0.707	3.151	0.08
SARA	0.030	2.748	0.915
Normal alleles	0.081	0.489	0.514
Expanded alleles	−0.159	1.778	0.376

*ATE, Average Tace Error; TTE, Test Time Execution; AAO, Age At Onset; SARA, The Scale for the Assessment and Rating of Ataxia.*

*Bold value showed significance.*

*^a^Male vs. female*.

**Table 4:** The influences of lower limb proprioception on disease severity.

	**Coefficient estimate**	**Standard error**	***P-*value**
**SARA**			
Gender^a^	−0.189	0.62	0.094
AAO	0.328	0.044	**0.019**
Normal alleles	0.099	0.044	0.374
Expanded alleles	0.565	0.137	**0.000**
ATE	0.036	0.016	0.800
TTE	−0.025	0.014	0.862

*ATE, Average Tace Error; TTE, Test Time Execution; AAO, Age At Onset; SARA, The Scale for the Assessment and Rating of Ataxia.*

*Bold value showed significance*

*^a^Male vs. female*.

## Discussion

To date, rehabilitation for SCA3 is a research hotspot, but there are few studies focusing on lower limb proprioception. Impaired lower limb proprioception can affect postural control and increase the risk of falling, which further aggravates the disease process of SCA3. Therefore, exploring the relevant influencing factors of proprioception and effectively formulating a corresponding rehabilitation plan are of great significance for the treatment of SCA3. The present study was the first to use the Pro-kin system to investigate lower limb proprioception in patients with SCA3 and explore the relationship between proprioception and clinical characteristics in patients.

Compared to the HC group, the patients with SCA3 exhibited significant impairments in lower limb proprioception. Our finding confirmed that cerebellar damage results in deficits of proprioception (12). Both groups have poor proprioception outcomes in their left limb compared with their right. This phenomenon may be explained that subjects of both groups are right-limb dominance, which was associate with better activation characteristics in the left primary sensorimotor cortex and the basal ganglia (22). Within the patient group, our regression analyses revealed that AAO and lengths of expanded CAG repeats predicted the severity of SCA3. Increasing AAO and disease duration predicted poorer lower limb proprioception. The correlations between disease duration and disease severity in SCAs and between greater functional loss and longer duration of the disease have been described (17, 23). Our findings are consistent with these research results. However, multivariable linear regression of SARA related to ATE and TTE showed no significant, so we failed to indicate that lower limb proprioception can predict the severity of ataxia in SCA3 patients.

In this work, we performed the lower limb proprioceptive function test instead of weakening somatosensory feedback by asking participants to stand on foam with both feet to analyze the interactions between postural stability and proprioceptive function (24). To the best of our knowledge, this study is the first to analyze the proprioception of the left and right feet separately in patients with SCA3. Our finding of significant impairments in lower limb proprioception represented by ATE and TTE measures using the Pro-kin system was supported by other studies on neurological disorders. For example, Sergio Bagnato found a significant increment of the ATE and TTE on the unaffected side leg in stroke patients as measured by the Pro-kin system (18). Patients with multiple sclerosis have also been reported to have worse assessment outcomes of ATE and TTE before proprioceptive training with the Pro-kin system (15).

Although the mechanism of how lower limb proprioception affects postural control in SCA3 is still poorly understood, there are several possibilities. First, patients present muscular rigidity and a locking of knees and ankles when undergoing testing for lower limb proprioception, causing abnormal joint movements related to postural control. The decline of knee and ankle joint position sense affects the implementation of knee and ankle strategies that are associated with postural control (25–28), which may be the cause of poor postural control function in SCA3 patients. Second, the pathological involvement of spinocerebellar proprioceptive input and the loss of integrity of the medial somatosensory descending system may explain the abnormal postural control (24, 29). Third, the cortical motor areas receive abnormalities in the peripheral afferent input or the brain response to sensory input may interfere with motor program processing in the cortical motor areas (18, 28).

This study is not without limitations. First, the force platform was limited to SCA3 patients who could safely perform the test (i.e. those able to stand unassisted for a certain duration). Second, as the study had a cross-sectional observational design and its evaluation of disease progression was retrospective, we did not provide information on changes in lower limb proprioception over time in our patients. Third, we did not use neurophysiological methods such as functional magnetic resonance imaging and somatosensory evoked potential. These methods are more specific to study the function of somatosensory descending system and cortical motor reaction, which could help indicate how lower limb proprioception affects postural control. Fourth, determining how to formulate targeted training of proprioception for SCA3 is worthy of further in-depth thinking.

## Conclusion

In this cross-sectional study, we showed that lower limb proprioception in patients with SCA3 was significantly impaired. Increasing AAO and disease duration were found to be related to impaired lower limb proprioception. However, lower limb proprioception could not predict the severity of ataxia in patients. These findings are important as they can help characterize the disease and thus assist in the development of new therapies and rehabilitation programs.

## Data Availability Statement

The original contributions presented in the study are included in the article/supplementary material, further inquiries can be directed to the corresponding author.

## Ethics Statement

The studies involving human participants were reviewed and approved by Ethics Committee of The First Affiliated Hospital, Fujian Medical University. The patients/participants provided their written informed consent to participate in this study.

## Author Contributions

X-HL: study concept and design, statistical analysis and interpretation, writing of the manuscript, and critical revision of the manuscript for important intellectual content. YL, H-LX, and AS: acquisition of data. JN and S-RG: study concept and design and acquisition of data. Z-YW: study concept and design, acquisition of data, analysis and interpretation, and critical revision of the manuscript for important intellectual content. All authors contributed to the article and approved the submitted version.

## Funding

This work was supported by the Startup Fund for Scientific Research, Fujian Medical University (Grant Number: 2019QH1095) to X-HL.

## Conflict of Interest

The authors declare that the research was conducted in the absence of any commercial or financial relationships that could be construed as a potential conflict of interest.

## Publisher's Note

All claims expressed in this article are solely those of the authors and do not necessarily represent those of their affiliated organizations, or those of the publisher, the editors and the reviewers. Any product that may be evaluated in this article, or claim that may be made by its manufacturer, is not guaranteed or endorsed by the publisher.
